# The Effect of β-Alanine Supplementation on Performance, Cognitive Function and Resiliency in Soldiers

**DOI:** 10.3390/nu15041039

**Published:** 2023-02-19

**Authors:** Ishay Ostfeld, Jay R. Hoffman

**Affiliations:** Department of Physical Therapy, School of Health Sciences, Ariel University, Ariel 40700, Israel

**Keywords:** military performance, dietary supplementation, ergogenic aid, carnosine, soldiers

## Abstract

β-alanine is a nonessential amino acid that combines with the amino acid histidine to form the intracellular dipeptide carnosine, an important intracellular buffer. Evidence has been well established on the ability of β-alanine supplementation to enhance anaerobic skeletal muscle performance. As a result, β-alanine has become one of the more popular supplements used by competitive athletes. These same benefits have also been reported in soldiers. Evidence accumulated over the last few years has suggested that β-alanine can result in carnosine elevations in the brain, which appears to have broadened the potential effects that β-alanine supplementation may have on soldier performance and health. Evidence suggests that β-alanine supplementation can increase resilience to post-traumatic stress disorder, mild traumatic brain injury and heat stress. The evidence regarding cognitive function is inconclusive but may be more of a function of the stressor that is applied during the assessment period. The potential benefits of β-alanine supplementation on soldier resiliency are interesting but require additional research using a human model. The purpose of this review is to provide an overview of the physiological role of β-alanine and why this nutrient may enhance soldier performance.

## 1. Introduction

Soldiers, especially those in special operational units, have physical capabilities that resemble those of competitive athletes and have been commonly referred to as a “*tactical athlete*” [1]. To perform their duties, soldiers must be highly fit to have the ability to run and react quickly with bursting power and speed, and travel on foot while carrying heavy loads for long distances [2,3]. Therefore, training as well as nutritional programs that are known to improve athlete performance would likely be of great benefit to the soldier [4]. Along with the physical characteristics, soldiers also require high cognitive function, often times making decisions under physical and mental stress and fatigue. Although the role of nutrition and dietary supplementation in improving physical performance has been extensively studied in both athletes and soldiers, the focus on psychological and cognitive performance improvements has lagged behind [5]. This review will specifically focus on the potential role that β-alanine supplementation has on soldier performance. A previous review by Hoffman et al. [6] that examined the effect of β-alanine on military performance was published several years ago. Since then, our knowledge of the potential benefits of β-alanine supplementation has grown, and additional work has been published on the efficacy of β-alanine on soldier performance. This review will provide an update on the work previously published and focus on the physiological role of β-alanine and how the use of this dietary ingredient can be used as a potential dietary intervention to enhance soldier performance, including both physical and cognitive function. In addition, the potential role that β-alanine may have on resiliency to specific issues common to the soldier combat experience, such as post-traumatic stress disorder (PTSD), mild traumatic brain injury (mTBI) and heat illness, will also be discussed.

## 2. Methodology and Search Strategy

To ensure that all relevant papers examining this topic were examined, the authors used search engines such as PubMed and Google Scholar using the terms β-alanine, soldiers, PTSD, mTBI, military, environmental extremes, and heat stress. For papers on human subjects to be included in the review, the research needed to be conducted on soldiers in the field or on participants performing a military simulated activity under controlled laboratory conditions. Animal studies were required to examine β-alanine as an intervention on a valid model of a military scenario such as PTSD, mTBI or heat stress.

## 3. Physiological Role of β-Alanine

β-alanine is a nonessential amino acid produced endogenously in the liver. In addition, humans acquire β-alanine through the consumption of foods such as meat and poultry [7,8]. β-alanine combines with the amino acid histidine to form the intracellular dipeptide carnosine, especially in type II skeletal muscle fibers [9,10], and is considered the rate-limiting step of carnosine synthesis [11,12]. β-alanine has only limited ergogenic effects by itself [13], and it appears that the ergogenic effects associated with β-alanine are related to the increase in carnosine content. 

Carnosine, by its hydrogen ion (H^+^) buffering capacity, has an important role in the maintenance of intracellular acid–base homeostasis [14,15,16], especially in humans [17]. Thus, by increasing carnosine content in skeletal muscle, β-alanine supplementation enhances intracellular buffering capacity, enabling a greater tolerance or delaying of muscle fatigue during sustained anaerobic activity [18,19,20]. This appears to be more efficacious during high-intensity activities that span 60–300 s in duration [21,22], in both men and women [23]. Additional physiological benefits associated with elevated carnosine content have been suggested to include potential elevated force production. As carnosine binds both H^+^ and Ca^2+^, the increase in H^+^ binding to carnosine may induce Ca^2+^ unloading at the level of the sarcomere, increasing cross-bridge formation and augmenting skeletal muscle force production [24,25]. In addition, several investigations have indicated that carnosine can act as an antioxidant, antiglycating and ion-chelating agent [26,27,28,29] by scavenging reactive oxygen species, superoxide anions and peroxyl radicals. Although β-alanine has been shown to be ineffective as an antioxidant itself [17,30,31], its ability to augment tissue carnosine content may result in an attenuation of the oxidative stress response, inflammation and muscle damage [32]. Carnosine has also been shown to act as an ion-chelating agent, preventing copper and zinc ions from excessive accumulation that may lead to lipid peroxidation and thus minimizing cellular damage [28]. Carnosine is also thought to act as an antiglycating agent, preventing the formation of advanced lipid peroxidation end-products and advanced glycoxidation end-products, delaying the aging process [17]. These additional roles attributed to elevated tissue carnosine levels have been primarily demonstrated in animal models, and evidence to support its role in humans still remains largely unknown [18]. 

In the past 10 years, evidence from animal models has indicated that β-alanine can also increase the carnosine content in various compartments in the brain and may serve as a neuroprotector [33,34,35]. These studies have shown that when β-alanine is supplemented prior to exposure to various stresses, it can increase the animal’s resiliency by maintaining brain-derived neurotrophic factor (BDNF) and attenuate the inflammatory response to the stressor. However, if β-alanine is supplemented in animals that are already experiencing inflammation, such as in aging, elevating brain carnosine levels from β-alanine supplementation does not appear to reduce brain inflammation or reverse learning dysfunction despite stimulating elevations in BDNF expression [36]. Considering that β-alanine is a dietary supplement and not a drug, it may be understandable that β-alanine can be effective in elevating any nutritional deficits, but it is likely unable to reverse the effects of an established inflammatory disease state or chronic inflammation. 

## 4. Dosing

Investigations demonstrating the efficacy of β-alanine supplementation have used a supplement regimen ranging from 1.6 to 12 g·day^−1^ and from 2 weeks to 6 months in duration [18]. These dosing protocols have resulted in significant increases in muscle carnosine content that have ranged from 23 to 200% from baseline levels [10,34,37,38,39]. Initial studies on β-alanine supplementation used doses of up to 6.4 g·day^−1^ due to the apparent greater risk of symptoms of paresthesia associated with higher doses [18]. However, the development of a sustained-release formulation of β-alanine has provided for a much greater daily dose to be used without the risk of paresthesia [40]. Elevations in muscle carnosine appear to be quite variable, which is likely related to several factors including initial muscle carnosine levels, dietary habits, type of exercise program, supplement dose and duration of supplementation. Supplementing with β-alanine results in significant and similar increases in carnosine content in both fast-twitch and slow-twitch muscle fibers [39,41]. This is despite a greater carnosine content observed in fast-twitch compared to slow-twitch fibers [9,10,42]. Increases in muscle carnosine are linearly related to β-alanine ingestion [21] and appear to follow first-order kinetics, in which a greater amount of β-alanine ingested results in a greater magnitude of muscle carnosine increase [43]. In opposition to the increase in muscle carnosine is an ongoing rate of decay, also reported to act as first-order kinetics [43]. Interestingly, in a study comparing daily 6 g and 12 g doses in college-age participants, the 12 g dose was able to result in a similar increase in muscle carnosine within 2 weeks compared to what was observed following 4 weeks of the 6-g·day^−1^ dose [37]. Although the higher dose appeared to provide a faster increase to achieve similar levels of muscle carnosine content, the total increase in muscle carnosine was not doubled, suggesting that a possible limitation may have occurred in either the transport of β-alanine, bioavailability of histidine or carnosine synthetase activity [43].

## 5. Efficacy of β-Alanine Supplementation

β-Alanine is considered one of the more popular supplements used by competitive athletes [38,44,45,46]. Supplementation with β-alanine is known to significantly enhance athletic performances during high-intensity prolonged activity [47,48,49,50]. The greatest ergogenic potential for β-alanine supplementation appears to be during high-intensity activity lasting 60–240 s in duration [8]. Although high-intensity activity lasting less than 60 s has a considerable anaerobic contribution, the highest level of acidosis occurs during high-intensity exercise lasting longer than 60 s [19].

## 6. β-Alanine Supplementation in Soldiers

Soldiers’ tactical performance includes a number of physical challenges that include prolonged runs and short sprints, lifting heavy loads such as carrying their gear on their back or wounded fellow soldiers, hand-to-hand combat and tactical shooting under physical stress. These physical challenges may also be required to be performed under cognitive stress with sleep deprivation [51]. Two studies have demonstrated the benefits of β-alanine supplementation (6 g·day^−1^ for 4 weeks) on soldiers’ tactical performance during high-intensity military activity [52,53]. During the study period in both investigations, soldiers from elite combat units were engaged in military training tasks that included combat skill development, physical work under pressure, navigational training, self-defense/hand-to-hand combat and conditioning. The results indicated that β-alanine supplementation was effective in maintaining lower body power (i.e., peak jump power) and psychomotor performance (i.e., target engagement speed and shooting accuracy following a weapon misfire) in soldiers that were supplemented with β-alanine compared to soldiers provided a placebo. Improvements were also reported in the 50 m casualty carry task and in cognitive function (i.e., mathematical calculations performed on an active live fire range) in soldiers supplementing with β-alanine. However, no changes were noted in the 2.5 km run, 1 min sprint or repeated sprints (5 × 30 m). Improvements in shooting performance were thought to be related to the effect of elevated muscle carnosine on enhancing fatigue resistance in muscle fibers by maintaining both standing and kneeling shooting positions as well as assisting the soldier in keeping the weapon steady during target acquisition and marksmanship [53]. However, later studies showing that β-alanine can cross the blood–brain barrier and increase carnosine levels in the brain [34,35], albeit in an animal model, suggest that improvements may also have been related to a possible reduction in oxidative stress associated with intense military training.

There has been only one study that has examined the effect of β-alanine supplementation on the inflammatory response to intense military training [54]. In this study, special operation soldiers were provided a high dose (12 g·day^−1^) of β-alanine for a week. The anti-inflammatory cytokine IL-10 was measured before and after two intense periods of training. IL-10 is an anti-inflammatory cytokine whose elevation is observed at the latter part of the inflammatory cascade [55]. The results of the study indicated that soldiers supplementing with β-alanine experienced a possibly greater increase in circulating IL-10 concentrations, suggestive of a potential therapeutic response to the highly intense military training. Soldiers had participated in two separate 5-day intense periods of military training that involved daily navigation of 27.8 km·day^−1^ and ~50% of their body mass in their packs. In addition, soldiers slept approximately 5 h per day only. The supplementation protocol occurred for the week between each of the intense training periods, which simulates a likely military scenario that deploys special operation personnel to multiple sustained operations interspersed with a brief recovery period [54]. 

## 7. β-Alanine and Soldiers’ Cognitive Function

The cognitive challenge of soldiers is no less important than their physical challenges, as soldiers from across military disciplines and special operation units are required to make decisions under conditions of fatigue, mental stress, major physical strain and sometimes under gunfire and explosions [56]. However, while some investigations have indicated possible cognitive benefits of β-alanine supplementation during highly intense military training [52] or simulated military training [57], other studies were unable to support that effect [53,58]. To the best of our knowledge, there have been only two studies conducted that examined the potential role of β-alanine on cognitive function in active soldiers [52,53] and an additional two studies that examined a simulated military operation conducted in a laboratory setting [57,58]. The field studies used special operation soldiers to examine both physiological and cognitive function. Soldiers’ cognitive performance was assessed by the serial subtraction test [59]. The test consists of a 2 min timed written test in which participants are required to subtract the number seven from a randomly generated four-digit number. The test measures how quickly and accurately the participant can compute a simple mathematical problem. The first time that this test was administered to soldiers who were supplementing with β-alanine, it was performed in a quiet area [53]. No differences were noted in performance between the soldiers who supplemented with β-alanine compared to the placebo. However, during that same study, tactical function was assessed by requiring the soldiers to recognize and fix a misfire during shooting and continue directing accurate fire on the target [53]. The improvements in accuracy and engagement speed demonstrated for the β-alanine group in comparison with the placebo group were either related to fatigue reduction or possibly to an improvement in cognitive decision-making ability. That study was the first to suggest possible cognitive benefits of β-alanine supplementation on soldiers’ tactical performances. This led to a second study by the same research team [52]. In that study, the serial subtraction test was again used as the cognitive assessment. However, in this study, it was performed within the shooting range while continuous live fire was being directed at stationary targets. This required the soldiers to maintain their focus despite the loud noise from the active firing line. This type of environment has been reported to increase levels of anxiety, making it difficult to solve mathematical problems and significantly decreasing cognitive performance in soldiers [60]. Soldiers ingesting β-alanine had a significantly greater number of correct answers on the 2 min test than soldiers who consumed the placebo. The differences between the two studies were the inclusion of the stress element. In the initial examination, the cognitive function measure was performed in a quiet area, while the second study used a highly stressful environment. Thus, the stress stimulus appeared to cause some potential change such that carnosine levels were able to provide a degree of resiliency, resulting in a maintenance of cognitive function.

There have been additional examinations that have simulated a military operation with sleep deprivation in a laboratory environment [58,61]. One study randomized 19 male college students (6 were former soldiers in the U.S. military and 10 participants were actively involved in the U.S. Army Reserve Training Corps) into β-alanine or placebo groups [61]. Following a 2-week supplementation period (12 g·day^−1^), participants performed a 24-h simulated military operation without any sleep permitted. During the 24-h period (from 8:00 am on day 1 until 8:00 on day 2), the participants were required to attend mission briefs, perform combat-specific activities, including participating in two, 2 h ruck marches, and were tested three times (at 0-h, 12-h and 24-h). A snack was provided as they reported to the laboratory, and a meal-ready-to-eat (MRE) was provided during the lecture-based training. Water was provided ad libitum throughout the 24-h period. Cognitive function assessments included the serial sevens test, several different reaction tests including one that provided a distraction to the subjects and a visual tracking assessment. The reaction test with distraction required the participant to react to a visual stimulus by striking it with their hand while verbally reciting a randomly generated five-digit number that was presented on an LCD screen of the testing apparatus. All testing was performed under controlled laboratory conditions. The results of the study revealed no significant group differences in mathematical processing, and no change from baseline was noted. Participants consuming the placebo had a slower visual reaction time at 24-h, while participants consuming β-alanine were able to maintain their visual reaction time throughout the study period. The reaction test with a distraction resulted in a significant decline in both groups by the end of the 24-h study, but no significant differences were noted between the groups in reaction performance [61]. However, the investigators also recorded the number of misses. Participants supplementing with β-alanine had a significantly lower number of misses compared to the placebo group. No differences between the groups were noted in visual tracking ability, and no changes from baseline were noted in either group. The authors concluded that β-alanine supplementation may have provided potential cognitive benefits [61]. Considering that during a sustained military operation, the ability to make decisions under stress and minimize mistakes in executive function is crucial to mission success, the results of that study provided interesting evidence supportive of β-alanine use in tactical athletes. 

Wells et al. [58] used the same simulated military operation and sleep deprivation protocol including the dosing regimen but used the automated neuropsychological assessment metrics (ANAMs) as a cognitive function assessment. Measurements occurred at 0-, 12-, 18- and 24-h. The ANAM measures a number of cognitive domains including processing speed, reaction time, working memory, short-term memory, spatial processing, sustained attention, computational skills, concentration and learning. In addition, it provides a measure of sleepiness and concussion symptoms as well. The results of the study showed that the level of sleepiness increased in both groups at both 18- and 24-h. However, no differences were noted between the groups. In addition, no between-group differences were noted in simple reaction time, code substitution, mathematical processing and a composite measure of cognitive function. Significant declines in cognitive function were noted as the level of sleepiness increased. However, β-alanine ingestion did not appear to provide any benefit for the participants in that investigation [58]. 

**Figure 1:**
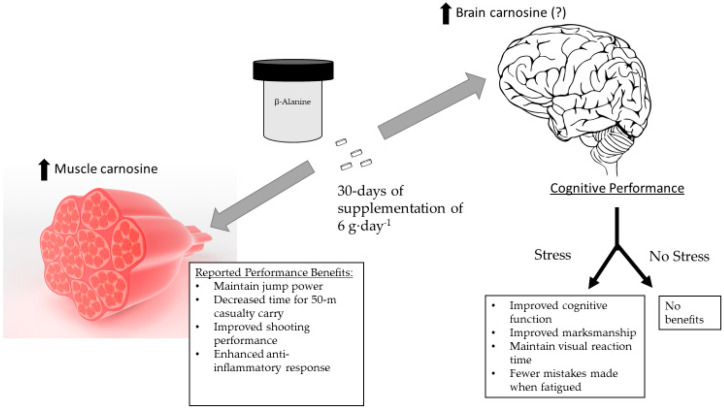
Physical performance and cognitive function changes subsequent to β-alanine supplementation in soldiers. Data from [52,53,54,58,61].

Supplementing with β-alanine appears to be beneficial for tactical athletes for both physical and cognitive function. However, the latter benefits may be realized only during elevated levels of stress. Figure 1 provides an overview of the benefits observed in studies examining human soldiers, primarily active-duty soldiers from special operation units. The question mark next to the elevations in brain carnosine is related to the lack of scientific evidence regarding elevated brain carnosine levels in human subjects resulting from β-alanine supplementation. To date, the only studies to show elevated brain carnosine levels have been reported in an animal model (see below). The lack of evidence is likely a function of technological limitations.

Interestingly, Loy and colleagues [62] have suggested that changes in cognition are influenced by both fatigue and energy but that they act independently. This is a scenario that may often impact military personnel during sustained operations, in which fatigue is quite high but the soldier can overcome the fatigue to an increase in energy. How β-alanine can impact this is not clear, but previous research has reported that β-alanine can modulate dopamine levels in the nucleus accumbens located in the basal forebrain [63]. However, there is very limited evidence to support this as a potential mechanism for β-alanine’s role in enhancing cognitive function during stressful conditions. Additional work should explore this possibility further. 

## 8. Post-Traumatic Stress Disorder and β-Alanine Supplementation

PTSD is a substantial cause of morbidity in soldiers and veterans [64,65]. Current treatment approaches are usually limited to post-event psychological support, while methods to build psychological resilience remain unclear. An animal study was the first to indicate that β-alanine supplementation was able to elevate carnosine levels in the brain, with a reported increase in BDNF expression and a decrease in 5-hydroxyindoleacetic acid (i.e., a metabolite of serotonin) in the hippocampus [33]. These changes were associated with reduced anxiety in the animals. Based upon these findings, Hoffman et al. [34] decided to examine the effect of β-alanine on PTSD. Rats were exposed to a predator scent stress (PSS) stimulus. The PSS has been previously demonstrated to be an effective model to determine the effects of various interventions on the behavioral response to stress [66,67]. The animals were provided β-alanine combined with glucomannan in their water for 30 days prior to exposure to the PSS. The glucomannan was used to slow absorption and act as a sustained-release formulation. The results of the study indicated that β-alanine ingestion in animals exposed to the PSS was effective at increasing resiliency. Animals that were fed β-alanine and exposed to the PSS had significantly lower anxiety measures than animals exposed and fed a normal diet. In addition, animals fed β-alanine experienced significant elevations in carnosine in the hippocampus, cortex, hypothalamus, amygdala and thalamus segments of the brain. These elevations were also associated with significant elevations in BDNF expression in the hippocampus. Elevations in BDNF expression and carnosine in the hippocampus were inversely correlated with the anxiety index (r = −0.471 and −0.550, *p* < 0.002, respectively). The data from this study provided initial evidence of the potential for β-alanine to increase resilience to PTSD [34]. However, it should be emphasized that this study did not use human models and β-alanine was given only preventively and prior to the stressful event. Further research is warranted in this area.

## 9. Mild Traumatic Brain Injury (mTBI) and β-Alanine Supplementation

Traumatic brain injury, and more specifically, mTBI, was the signature wound emanating from Operation Enduring Freedom [68]. mTBI is a nonpenetrating head injury that is often the result of a soldier being in close proximity to a low-pressure blast wave. mTBI is characterized by elevations in oxidative stress and inflammation in the brain, which is also part of the sequelae of physiological events contributing to PTSD [69]. These changes may also contribute to the cognitive impairment and neurodegeneration associated with mTBI [70]. A study to examine the effect of β-alanine supplementation on an animal model of mTBI was conducted [35], using a similar supplementation protocol as previously described [34]. Animals were provided either 30 days of β-alanine supplementation consumed with their normal diet or were provided a normal diet only. Animals were then exposed to a low-pressure blast wave. Exposure to a low-pressure blast wave has been shown to be a valid animal model to elicit distinct behavioral and morphological changes that simulate mTBI-like, PTSD-like and comorbid mTBI-PTSD-like responses [71]. The method of explosion used in the study produced a cylindrical blast wave that simulated a blast wave profile similar to that seen from an explosive device common to the battlefield. In an actual explosion, the blast wave causes an acute, short-duration elevation in pressure followed by a negative phase. The animals were not anesthetized and were subjected to a single blast wave with their head facing the blast without any body shielding, resulting in a full body exposure to the blast wave. This blast protocol resulted in a sound pressure level of 193 dB and a light intensity of approximately 5 Mlux [71], which is similar to that experienced during exposure to an M84 stun grenade at a distance of 1.5 m (3.1 Mlux). The results indicated that β-alanine supplementation was effective in reducing the incidence of the mTBI-like phenotype following exposure to the blast [35]. Of the animals that were exposed to the blast wave, 46% of the animals that were provided a normal diet exhibited mTBI symptoms, while only 23.5% of animals supplemented with β-alanine exhibited mTBI symptoms [35]. These differences were statistically different (*p* = 0.044). These results provided further evidence that β-alanine supplementation may increase resilience to nonpenetrating battlefield injuries. Although the results of this study may have great relevance for soldiers, it should be emphasized that this study did not use human models and β-alanine was given only preventively and prior to the blast event. 

## 10. β-Alanine Supplementation and Exposure to Heat Stress

Exertional heatstroke is a typical injury in competitive athletes as well as in soldiers and a common cause of death among military recruits during training [72,73]. A recent study examined whether β-alanine supplementation can provide a degree of resiliency to animals exposed to heat stress [74]. Animals were provided with 30 days of β-alanine mixed with glucomannan in an 80:20 ratio at the same dose (100 mg·kg^−1^). A total of 30 mg of powder was dissolved in 25 mL of water. This supplement protocol has been demonstrated in several studies to significantly increase brain carnosine levels [34,35]. Following the supplementation protocol, animals were exposed to heat stress. Animals were exposed for 2 h to a passive heat stress protocol that was conducted in a reach-in climatic chamber. The protocol has been previously validated [75] and requires the animals to be exposed to a constant temperature of 40–41 °C. Animals that were supplemented with β-alanine experienced an attenuated thermoregulatory response to the acute heat exposure. Exposure to the heat stress resulted in significant reductions in BDNF and neuropeptide Y, suggesting that heat stress can result in a potential decrease in neuronal plasticity during heat stress. Animals supplemented with β-alanine appeared to maintain BDNF expression in the Cornu Ammonis 3 region (CA3) of the hippocampus and the paraventricular nucleus of the hypothalamus. In addition, animals supplemented with β-alanine also had an attenuated inflammatory response and a lower thermoregulatory stress compared to animals fed a normal diet. The results suggest that β-alanine supplementation prior to exposure to heat stress may attenuate the inflammatory response, maintain neurotrophin and neuropeptide levels and potentially increase the survival rate during heat stress [74].

## 11. Safety

The only side effect associated with β-alanine supplementation is paresthesia. Paresthesia is a sensation of numbing or tingling in the skin. Symptoms of paresthesia generally disappear within 60–90 min following supplementation [21]. Paresthesia is generally seen when individuals ingest β-alanine in doses exceeding 800 mg·kg^−1^ in a non-sustained-release form [12]. In a study using sustained-release formulations, no differences were noted in symptoms of paresthesia between participants consuming the placebo and participants consuming the supplement, even at a daily dose of 12 g of sustained-release β-alanine (four doses of 3 g·day^−1^) [37]. The mechanism that is thought to be responsible for paresthesia involves the binding of β-alanine to Mas-related G-protein-coupled receptor D (MrgprD) [18]. MrgprD is expressed in cutaneous sensory neurons in the skin, and the binding of β-alanine to MrgprD has been suggested to be responsible for the associated symptoms of paresthesia [76,77]. 

The highest reported daily dose of β-alanine provided has been 12 g·day^−1^ [18,57,58,61] for two weeks. This dosing protocol resulted in no change in any of the hematological variables measured, and all blood count variables remained within normal ranges during the supplementation period [18]. Harris et al. [12] reported no change on a 12-lead electrocardiogram or in any hematological markers following 4 weeks of 3.2 g·day^−1^ of β-alanine ingestion. In a study examining the effect of 24 weeks of β-alanine supplementation (6.4 g·day^−1^), no changes were noted in any blood markers of renal, hepatic and muscle function [78]. A recent meta-analysis concluded that β-alanine supplementation within the doses reported in numerous investigations does not adversely affect those individuals consuming it [79].

## 12. Conclusions

This review focused on investigations examining β-alanine supplementation and soldier performance. Based on the evidence available, the efficacy in regard to β-alanine supplementation and physical performance in soldiers is similar to what is generally seen in competitive athletes. Evidence exploring the effect of β-alanine and cognitive function is inconclusive, but may be more of a function of the stressor that is applied during the assessment period. It is also possible that the benefits of β-alanine supplementation may be focused on specific job tasks. For instance, snipers and breachers are required to focus on a “narrow target”, while commanders are required to have a “broad vision”. Further research is warranted in this area. Finally, the evidence examining the ability of β-alanine to increase resilience to common stressors found on the battlefield is exciting but has been demonstrated in an animal model only. Additional research is warranted to provide further support, and the use of a human model is important.

## Data Availability

Not applicable.
